# Inhibition of central activation of the diaphragm: a mechanism of weaning failure

**DOI:** 10.1152/japplphysiol.00856.2019

**Published:** 2020-07-16

**Authors:** Franco Laghi, Hameeda Shaikh, Stephen W. Littleton, Daniel Morales, Amal Jubran, Martin J. Tobin

**Affiliations:** ^1^Division of Pulmonary and Critical Care Medicine, Hines Veterans Affairs Hospital, Hines, Illinois; ^2^Division of Pulmonary and Critical Care Medicine, Loyola University Chicago Stritch School of Medicine, Maywood, Illinois

**Keywords:** diaphragm, mechanical ventilation, muscle fatigue, neuromuscular coupling, weaning from mechanical ventilation

## Abstract

During a T-tube trial following disconnection of mechanical ventilation, patients failing the trial do not develop contractile diaphragmatic fatigue despite increases in inspiratory pressure output. Studies in volunteers, patients, and animals raise the possibility of spinal and supraspinal reflex mechanisms that inhibit central-neural output under loaded conditions. We hypothesized that diaphragmatic recruitment is submaximal at the end of a failed weaning trial despite concurrent respiratory distress. Tidal transdiaphragmatic pressure (ΔP_di_) and electrical activity (ΔEA_di_) were recorded with esophago-gastric catheters during a T-tube trial in 20 critically ill patients. During the T-tube trial, ∆EA_di_ was greater in weaning failure patients than in weaning success patients (*P* = 0.049). Despite increases in ΔP_di_, from 18.1 ± 2.5 to 25.9 ± 3.7 cm H_2_O (*P* < 0.001), rate of transdiaphragmatic pressure development (from 22.6 ± 3.1 to 37.8 ± 6.7 cm H_2_O/s; *P* < 0.0004), and concurrent respiratory distress, ∆EA_di_ at the end of a failed T-tube trial was half of maximum, signifying inhibition of central neural output to the diaphragm. The increase in ΔP_di_ in the weaning failure group, while ∆EA_di_ remained constant, indicates unexpected improvement in diaphragmatic neuromuscular coupling (from 46.7 ± 6.5 to 57.8 ± 8.4 cm H_2_O/%; *P* = 0.006). Redistribution of neural output to the respiratory muscles characterized by a progressive increase in rib cage and accessory muscle contribution to tidal breathing and expiratory muscle recruitment contributed to enhanced coupling. In conclusion, diaphragmatic recruitment is submaximal at the end of a failed weaning trial despite concurrent respiratory distress. This finding signifies that reflex inhibition of central neural output to the diaphragm contributes to weaning failure.

**NEW & NOTEWORTHY** Research into pathophysiology of failure to wean from mechanical ventilation has excluded several factors, including contractile fatigue, but the precise mechanism remains unknown. We recorded transdiaphragmatic pressure and diaphragmatic electrical activity in patients undergoing a T-tube trial. Diaphragmatic recruitment was submaximal at the end of a failed trial despite concurrent respiratory distress, signifying that inhibition of central neural output to the diaphragm is an important mechanism of weaning failure.

## INTRODUCTION

Critically ill patients who fail a trial of weaning from mechanical ventilation (6, 8) do not develop contractile fatigue of the diaphragm (55). Attempting to shed light on this phenomenon, investigators have conducted studies in patients with respiratory disorders (25, 57, 78, 89), healthy volunteers (26, 58), and in animals (96). Findings in these studies raise the possibility of spinal and supraspinal reflex mechanisms that inhibit central neural output under loaded conditions (39, 58, 104). These reflex mechanisms may have a protective action against load-induced muscle damage (39, 58, 104).

To date, it is not known whether (or not) patients who fail a weaning trial experience a reflex inhibition of central activation. This gap in our knowledge stems from the difficulty in assessing the extent of diaphragmatic recruitment during weaning (55). Recruitment can be quantified by performing the interpolated twitch pressure technique, whereby a large increment in transdiaphragmatic pressure (P_di_) elicited by phrenic nerve stimulation superimposed upon perceived maximal voluntary inspiratory efforts signifies incomplete muscle recruitment and, thus, reflex inhibition of central activation (39). During weaning, it is virtually impossible to assess diaphragmatic recruitment using twitch interpolation (55) because the technique requires precise timing of phrenic nerve stimulation at the zenith of an inspiratory effort (that is maintained steady during stimulation) and also supramaximal recruitment of the phrenic nerve throughout the stimulation (39). In addition to being technically demanding (34), the interpolation technique is limited by its insensitivity to changes in diaphragmatic motor-unit firing rate (11, 97), an important component of central activation (39, 97, 104).

Limitations of the twitch-interpolation technique for assessing the presence of reflex inhibition of central neural output during weaning can be overcome by comparing electrical activity of the crural diaphragm during weaning against electrical activity during maximal inspiratory efforts (10, 48, 97). This technique relies on the use of multipolar esophageal electrodes and on sophisticated online processing of electrical signals originating from the crural diaphragm (97). This system allows quantification of diaphragmatic recruitment even during nonisometric contractions (11, 48), such as inhalation during weaning.

The objective of the current study, conducted in patients deemed ready for a trial of weaning from mechanical ventilation, was to compare, for the first time, the extent of diaphragmatic recruitment during weaning. Specifically, we hypothesized that diaphragmatic recruitment is submaximal at the end of a failed weaning trial despite concurrent respiratory distress.

## METHODS

### Patients

This prospective physiological study was conducted in the intensive care units (ICU) of the Hines VA Hospital, Hines, IL. Twenty critically ill male patients who were receiving mechanical ventilation through a cuffed endotracheal (*n* = 16) or tracheostomy tube (*n* = 4) and whose primary physician considered them ready to undergo a trial of weaning were enrolled in the study **(**Table 1). The patients had received 12 ± 3 (SE) days of ventilator support. The investigation was approved by the local Human Studies Subcommittee, and informed consent was obtained from each patient or authorized surrogate.

**Table 1.** Patient characteristics

Patient No.	Age, yr	Diagnosis	Airway	Days of Ventilator Support
Weaning success				
1	80	B cell lymphoma, tumor lysis syndrome, cardiac arrest	ET	2
2	68	Nonspecific interstitial pneumonia	ET	10
3	71	Sepsis, aortic stenosis, COPD	ET	21
4	40	Hypersensitivity pneumonitis	ET	10
5	56	Aspiration pneumonia, incomplete C5–7 spinal cord injury with syringomyelia	Trach*	5
6	64	Postoperative respiratory failure	ET	1
7	65	COPD, hypercapnic respiratory failure, seizure	ET	4
				
Weaning failure				
1	78	Small bowel obstruction, COPD	ET	4
2	56	Small cell lung cancer, neutropenic fever	Trach	21
3	58	Sepsis, moderate leg-muscle deficit secondary to intracranial hemorrhage	Trach	44
4	60	Squamous cell lung cancer	ET	5
5	53	Alcohol withdrawal, pneumonia	ET	21
6	59	Postoperative respiratory failure, COPD	ET	3
7	85	Septic shock, coronary artery disease	ET	8
8	69	Septic shock, small bowel obstruction, COPD	ET	22
9	65	Cardiac arrest, abdominal aortic aneurysm	ET	11
10	63	Aspiration pneumonia, COPD	ET	9
11	84	Postoperative respiratory failure	ET	2
12	62	Hemorrhagic shock, COPD	ET	7
13	67	Septic shock, pneumonia	Trach	34

COPD, chronic obstructive pulmonary disease; ET, endotracheal tube; Trach, tracheostomy tube.

* Tracheostomy placed 7 yr previously for treatment of obstructive sleep apnea.

### Experimental Setup

#### Flow and pressure measurements.

Flow was measured with a heated Fleisch pneumotachograph placed between the endotracheal tube and the Y-piece of the ventilator circuit (Hans Rudolph, Kansas, MO) (55). Airway pressure (P_aw_) was measured proximal to the endotracheal tube. Esophageal (P_es_) and gastric pressures (P_ga_) were measured with balloon-tipped catheters coupled to pressure transducers (54). Proper positioning of the esophageal balloon catheter was ensured with the occlusion technique (55). Transdiaphragmatic pressure (P_di_) was obtained by subtracting P_es_ from P_ga_ (54).

#### Electrical activity of the diaphragm.

The electrical activity of the crural diaphragm, a reliable reflection of neural output to the diaphragm (46, 68) and diaphragmatic activation (12, 13, 97, 99, 101), was recorded with nine stainless-steel electrodes mounted on a polyurethane tube positioned across the gastroesophageal junction and wired as eight overlapping bipolar pairs (NeuroVent Research, Toronto, ON, Canada) (58). All signals were recorded continuously and were processed using the method of Sinderby et al. (97). For each patient, these signals were normalized to the maximum change in electrical activity of the crural diaphragm recorded during the entire experiment in that patient (109). Throughout the report, these normalized signals are referred as EA_di_ (100).

### Protocol

The purpose of this experiment was threefold: to measure the extent of diaphragmatic recruitment at the conclusion of a T-tube trial, to examine diaphragmatic neuromuscular coupling during the trial, and to determine the mechanisms modulating neuromuscular coupling during a T-tube trial. Sedation was held before weaning, and patients were confirmed to be responsive (25). The Richmond Agitation-Sedation Scale was 0 (alert and calm) in 19 patients and −1 (drowsy) in one patient.

Following placement of all transducers, endotracheal suctioning was performed. Thereafter, to record passive mechanics of the respiratory system, controlled ventilation was achieved by instructing patients to relax, while the back-up rate on the ventilator was gradually increased until the patient's inspiratory muscle activity was suppressed (55). Tidal volume (600 ± 20 mL), inspiratory flow (64 ± 2 L/min), positive end-expiratory pressure (1.6 ± 0.5 cm H_2_O), and the set fractional concentration of inspired oxygen (0.40 in all instances) were kept constant. The airway opening was then occluded at the end of a passive inflation for a duration sufficient to achieve a plateau in airway pressure (P_aw_) (55).

Patients were then disconnected from the ventilator and maximum inspiratory airway pressure, maximum inspiratory transdiaphragmatic pressure, and concurrent electrical activity of the diaphragm were measured during a 20-s occlusion of the airway (55). Patients were then placed back on the ventilator for at least 2–3 min, while the T-tube system for the weaning trial was set up. Next, patients were disconnected from the ventilator and began to breathe spontaneously through the T-tube circuit with oxygen delivered at the same concentration as during mechanical ventilation; applied positive end-expiratory pressure was zero in all patients. The trial was continued for up to 1 h as tolerated (55). The a priori criteria for termination of the trial were development of respiratory distress (113), including tachypnea, facial sign of distress, diaphoresis, increased sternomastoid activity, hypoxemia (oxygen saturation <90% with a fraction of inspired oxygen ≥0.4), or development of tachycardia, hypotension, hypertension, or new arrhythmias (55, 113). Patients who met these criteria were returned to the ventilator and designated as weaning-failure patients. Patients who met none of these criteria at the end of the trial were extubated and they were designated as weaning-success patients. Throughout data acquisition, patients were studied while lying at 30° with their neck in the neutral position.

### Physiological Measurements

#### Respiratory mechanics.

Inspiratory resistance and elastance of the respiratory system before weaning and inspiratory resistance and dynamic elastance of the lung during weaning were calculated as previously described (55, 82). (In one success and in three failure patients, we had incomplete resistance and elastance data during weaning because of malfunction of the flow transducer.)

#### Expiratory muscle recruitment.

Expiratory muscle recruitment was measured as the increase in P_ga_ over the course of expiration (61, 79).

#### Intrinsic positive end-expiratory pressure.

Intrinsic positive end-expiratory pressure (PEEP_i_) was measured during spontaneous breathing as the negative deflection in P_es_ between the onset of inspiratory effort (end-expiratory P_es_) and the onset of inspiratory flow (55). Relaxation of the abdominal muscles at the onset of inspiration can contribute to the fall in P_es_ at the onset of inspiratory effort (79). Accordingly, any increase in P_ga_ over the course of the preceding exhalation was subtracted from the P_es_ signal (79).

#### Tension-time index of the diaphragm.

Tension-time index of the diaphragm (TTdi) (58), an estimate of diaphragmatic inspiratory effort relative to diaphragmatic strength, was calculated as the product of mean inspiratory P_di_ (normalized for P_di_max) and fractional inspiratory time.

#### Ratio of swings in gastric pressure to swings in esophageal pressure.

The relative contribution of the rib cage and expiratory muscles to tidal breathing was assessed as the ratio of swings in P_ga_ to swings in P_es_ (ΔP_ga_-to-ΔP_es_ ratio). ΔP_es_ was measured from the beginning of effort to its nadir. ΔP_ga_ was measured from the beginning of effort (also identified from the P_es_ tracing) to its maximum excursion (45).

#### Rate of rise in P_di_ and rate of rise in EA_di_.

The rate of transdiaphragmatic pressure development during inhalation was assessed as the ratio of swings in P_di_ to contraction time (ΔP_di_/T_I_) (58, 78) (Supplemental Fig. S1, https://doi.org/10.6084/m9.figshare.11903193). The rate of phrenic motor-neuron activation (reflecting both motor unit recruitment and motor unit firing rate) was assessed as the ratio of swings in EA_di_ (ΔEA_di_) to contraction time (ΔEA_di_/T_I_) (14, 33, 41, 65) (Supplemental Fig. S1, https://doi.org/10.6084/m9.figshare.11903193).

#### Diaphragmatic neuromuscular coupling.

Neuromuscular coupling of the diaphragm was assessed as the ratio of tidal change in transdiaphragmatic pressure (ΔP_di_) to tidal change in the electrical activity of the crural diaphragm (ΔEA_di_) (25, 58), with the latter normalized to the maximum ΔEA_di_ recorded during the entire experiment (109). (For ΔP_di_/ΔEA_di_ ratio to represent the ability of the muscle to convert a given neural output into pressure, ΔP_di_ must approximate the force output of the diaphragm during a given contraction, and ΔEA_di_ must approximate the neural output to the diaphragm during that given contraction—both conditions have been validated under a variety of experimental conditions (10, 18, 29, 33, 41, 46, 53, 65, 68, 69).).

#### Central neural output to the diaphragm.

Central neural output to the diaphragm was defined as the amplitude of diaphragmatic electrical activity (∆EA_di_) (7, 9, 25, 27, 47, 63, 97, 106, 109). Reflex inhibition of central neural output during weaning was defined, in operational terms, as submaximal diaphragmatic recruitment in the presence of respiratory distress (58). The extent of diaphragmatic recruitment at the conclusion of a weaning trial was quantified as the ratio of the mean tidal change in diaphragmatic electrical activity recorded during the last 30 to 60 s of breathing through a T-tube circuit to the maximum ΔEA_di_ recorded during the entire experiment in that patient.

### Statistical Analysis

Baseline characteristics of patients with a successful and unsuccessful weaning outcome were compared using unpaired *t* tests. For the weaning trial, the data were analyzed at five points in time: the first and last minute of the trial and three periods taken at equal time intervals between the first and last minute. Mean data were calculated on the basis of 30- to 60-s recordings at the five points.

Data at the different time periods were compared by ANOVA for repeated measures when performing within-group comparisons, and mixed ANOVA when performing between-group comparisons. Statistical tests were two-sided, and *P* ≤ 0.05 was considered significant. All statistical evaluations were performed using SPSS 23 (IBM SPSS, Armonk, NY).

## RESULTS

Thirteen patients failed the weaning trial after 33 ± 6 min of breathing through the T-tube apparatus and mechanical ventilation was reinstituted. All patients who failed the weaning trial did so because of respiratory distress; no patient failed because of cardiovascular instability. Seven patients tolerated the T-tube trial for 60 min without distress; six were extubated, and one patient with a chronic tracheostomy (for sleep apnea) remained disconnected from the ventilator.

### Mechanics and Diaphragmatic Strength and Recruitment before the T-Tube Trial

Before the T-tube trial, passive respiratory system mechanics were not different between the success and failure patients (Table 2). P_di_max, ∆EA_di_, and diaphragmatic neuromuscular coupling during the maximal occlusion maneuvers were also equivalent in the success and failure patients (Table 2).

**Table 2.** Inspiratory muscle strength, electrical activity of the diaphragm, and respiratory mechanics before the T-tube trial

	Weaning Success (*n* = 7)	Weaning Failure (*n* = 13)	*P*
P_aw_max, cm H_2_O	−49.1 ± 4.9	−48.1 ± 4.8	0.891
P_di_max, cm H_2_O	45.4 ± 6.5	43.5 ± 4.2	0.799
∆EA_di_ (during P_di_max) %	77.3 ± 8.8	74.3 ± 4.3	0.735
P_di_max/∆EA_di_, cm H_2_O/%	63.5 ± 10.3	60.2 ± 6.4	0.777
Rrs, cm H_2_O·L^−1^·s	19 ± 4	23 ± 2	0.235
Ers, cm H_2_O/L	30 ± 3	30 ± 3	0.921
PEEPi, cm H_2_O	2.7 ± 1.6	5.7 ± 1.3	0.111

Values are expressed as means ± SE. Ers, elastance of the respiratory system; P_aw_max, voluntary maximal inspiratory airway pressure; P_di_max, voluntary maximal inspiratory transdiaphragmatic pressure; Rrs, inspiratory resistance of the respiratory system; PEEPi, positive end expiratory pressure; ΔEA_di_, change in the electrical activity of crural diaphragm during the P_di_max maneuver normalized to the maximum change in EA_di_ recorded during the entire experiment.

### Recruitment, Coupling, and Pressure Output During the T-Tube Trial

**Fig. 1.**
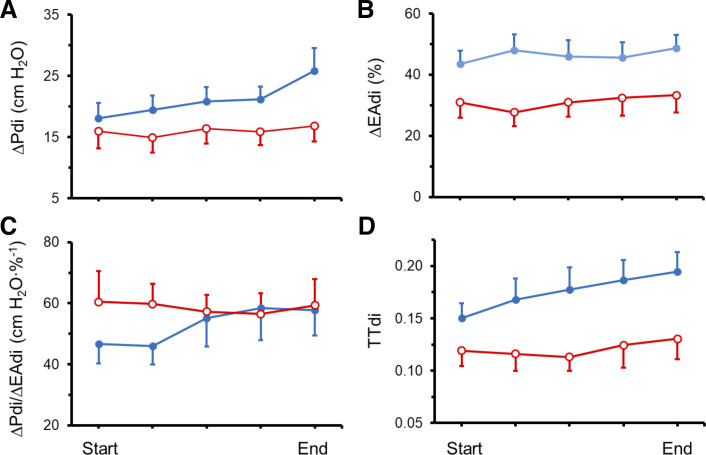
Tidal change in transdiaphragmatic pressure (ΔP_di_; *A*), diaphragmatic electrical activity (ΔEA_di_; *B*), neuromuscular coupling (ΔP_di_/ΔEA_di_; *C*) and tension-time index of the diaphragm (TTdi; *D*) during the course of a T-tube trial in weaning failure patients (solid blue circles; *n* = 13) and weaning success patients (open red circles; *n* = 7). Between onset and end of the trial, increases in ΔP_di_, ΔP_di_/ΔEA_di_, and TTdi occurred in the weaning failure group (*P* < 0.010), but not in the weaning success group. Over the course of the trial, weaning failure patients had higher values of ΔEA_di_ (*P* = 0.049) and tended to have a higher TTdi (*P* = 0.053) than did weaning success patients (see text for details). Data are presented as means ± SE and were analyzed by ANOVA.

From start to end of the T-tube trial, ∆EA_di_ was greater in the failure group than in the weaning success group (*P* = 0.049) (Fig. 1). In both weaning-failure and weaning-success patients, mean ∆EA_di_ at the end of the trial was less than the patients’ maximum ∆EA_di_ (*P* < 0.0005).

Clinical evidence of severe respiratory distress was confined to the weaning-failure patients. Despite these patients generating higher values of ∆EA_di_ (contrasted with behavior of weaning-success patients, *P* = 0.047) at the end of the trial, ∆EA_di_ was half of maximum in the failure group (48.6 ± 4.4%). The presence of submaximal diaphragmatic recruitment despite severe respiratory distress signifies reflex inhibition of central neural output to the diaphragm in the weaning-failure patients.

At the start of the T-tube trial, ∆P_di_ was equivalent in the two groups (Fig. 1). Between onset and end of the trial, ∆P_di_ increased in the failure group (*P* < 0.001), while it did not change in the success group (Supplemental Table S1, https://doi.org/10.6084/m9.figshare.12298340.v1). At end of the trial, ∆P_di_ was two-thirds of P_di_max (58.8 ± 5.2%) in the failure group and less than half of P_di_max (38.7 ± 5.3%) in the success group (*P* = 0.023).

The constant ∆EA_di_ in the two patient groups during the T-tube trial and the progressive increase in ∆P_di_ only in the failure group had two consequences. Coupling (∆P_di_/∆EA_di_) increased in the failure group between the onset and end of the trial: 46.7 ± 6.5 to 57.8 ± 8.4 cmH_2_O/%; *P* = 0.006 (Fig. 1). In contrast, coupling did not change between onset and end of the trial in the success group: 60.5 ± 10.1 to 59.4 ± 8.6 cm H_2_O/%.

Compared with ∆P_di_/∆EA_di_ during maximal inspiratory efforts recorded before the T-tube trial, ∆P_di_/∆EA_di_ worsened at the start of the trial in the failure group (*P* = 0.009) and remained unchanged in the success group (Table 2 and Fig. 1). As the trial progressed, the increase (improvement) in coupling in the failure group was such that the value of ∆P_di_/∆EA_di_ at the end of the trial was not different from ∆P_di_/∆EA_di_ recorded during maximal inspiratory efforts before the trial. This increase in coupling in the failure group was closely related to increase in mechanical load on the respiratory muscles: the *r*^2^ between coupling and inspiratory resistance during the failed trial was 0.889 (*P* = 0.016) (see *Breath Components and Respiratory Mechanics During the T-Tube Trial*). Between the onset and the end of the trial, TTdi increased in the failure group (*P* < 0.010), while it did not change in the success group (Fig. 1).

**Fig. 2.**
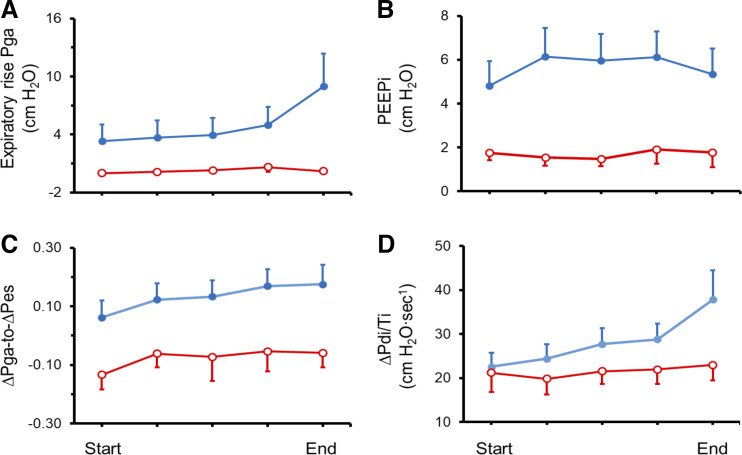
Expiratory rise in gastric pressure (P_ga_), evidence of expiratory muscle recruitment during exhalation (*A*), intrinsic positive end-expiratory pressure (PEEP_i_; *B*), ratio of tidal change in gastric pressure to tidal change in esophageal pressure (ΔP_ga_-to-ΔP_es_), an index of rib-cage and expiratory muscle contribution to respiratory effort (*C*), and rate of transdiaphragmatic pressure development (ΔP_di_/T*_I_*) (*D*) during the course of a T-tube trial in failure patients (solid blue circles; *n* = 13) and success patients (open red circles; *n* = 7). Between onset and end of the trial, expiratory rise in P_ga_, ΔP_ga_-to-ΔP_es_ ratio and ∆P_di_/T_I_ increased in failure group (*P* ≤ 0.031) and did not change in the success group. During the trial, expiratory rise in P_ga_, PEEP_i_, and ∆P_ga_-to-∆P_es_ ratio were greater in the failure group than in the success group (*P* < 0.025). The increases in P_ga_, ΔP_ga_-to-ΔP_es_ could have contributed to the improved neuromuscular coupling during the trial (see text for details). Data are presented as means ± SE and analyzed by ANOVA.

∆P_di_/T_I_ (rate of transdiaphragmatic pressure development) in the success group did not change between the onset and end of the trial: 21.2 ± 4.4 to 22.9 ± 3.5 cm H_2_O/s. In contrast, ∆P_di_/T_I_ increased between onset and end of the trial in the failure group: 22.6 ± 3.1 to 37.8 ± 6.7 cm H_2_O/s (*P* < 0.0004) (Fig. 2). ΔEA_di_/T_I_ (estimate of rate of phrenic motor neuron activation) in the success group did not change between the onset and end of the trial: 40.7 ± 8.0 to 46.5 ± 9.6%/s. In contrast, ΔEA_di_/T_I_ tended to increase between onset and end of the trial in the failure group: 52.1 ± 4.8 to 66.4 ± 6.2%/s (*P* = 0.056). Between onset and end of the trial, failure patients experienced a greater increase in ΔP_di_/T_I_ (67.1%) than in ΔEA_di_/T_I_ (36.1%, *P* < 0.006). Improvement of neuromuscular coupling between onset and end of the failed trial (Fig. 1) was the likely mechanism for the greater increase in ΔP_di_/T_I_ than in ΔEA_di_/T_I_.

### PEEPi, Rib Cage, and Expiratory Muscle Recruitment During the T-Tube Trial

During the T-tube trial, PEEP_i_ and expiratory rise in P_ga_ (estimate of the magnitude of expiratory muscle recruitment) were greater in failure patients than in success patients (*P* < 0.025 in both instances) (Fig. 2). Similarly, during the T-tube trial, the ∆P_ga_-to-∆P_es_ ratio, an index of rib cage and expiratory muscle contribution to respiratory effort, was greater in the failure group than in the success group (*P* = 0.024) (Fig. 2).

### Breath Components and Respiratory Mechanics During the T-Tube Trial

At the onset of the trial, the success and failure groups had equivalent readings of respiratory frequency (23.7 ± 1.7 and 26.5 ± 2.1 breaths/min), tidal volume (0.367 ± 0.034 and 0.397 ± 0.059 L), inspiratory resistance of the lung (12.0 ± 1.0 and 19.5 ± 3.3 cm H_2_O·L^−1^·s), and dynamic elastance of the lung (22.4 ± 4.4 and 17.9 ± 3.3 cm H_2_O/L).

Between onset and end of the trial, respiratory frequency, inspiratory resistance, and dynamic elastance of the lung increased in the failure group (*P* < 0.05) and did not change in the success group; tidal volume did not change in either group. During the T-tube trial, inspiratory resistance was greater in the failure group than in the success group (*P* = 0.022).

## DISCUSSION

This investigation has three major novel findings. The extent of diaphragmatic coupling during maximal voluntary inspiratory maneuvers recorded before the T-tube trial was similar in weaning failure and success groups. Compared with equivalent coupling during maximal maneuvers before the trial, coupling initially worsened in the failure group during the T-tube trial but then improved, whereas it did not change in the success group throughout the trial. Despite an increase in neural output to the respiratory muscles and development of respiratory distress at the end of the failed weaning trial, diaphragmatic recruitment was submaximal; this finding signifies inhibition of central neural output to the diaphragm.

### Diaphragmatic Coupling before the T-Tube Trial

The failure and success groups had equivalent levels of pressure output, neural activation, and neuromuscular coupling of the diaphragm (ΔP_di_/∆EA_di_) during maximal voluntary inspiratory maneuvers before the T-tube trial. These results imply that diaphragmatic weakness, unless extreme, is not the mechanism responsible for weaning failure. This concurs with our recent observation in 169 patients managed in a long-term acute-care hospital who were successfully detached from the ventilator (49). Maximal voluntary inspiratory pressure did not change between admission and discharge (45.4 ± 1.3 vs. 48.1 ± 1.3 cm H_2_O) (49). Our results also clarify why increases in maximal inspiratory pressure following strength training do not necessarily guarantee weaning success (19, 72) or reduce reintubation rates (15).

### Diaphragmatic Coupling and Diaphragmatic-Pressure Output during the T-Tube Trial

In contrast with equivalence of coupling in the two groups before the trial, coupling worsened initially in the weaning failure patients during the T-tube trial. Shortening of diaphragmatic fibers consequent to hyperinflation was the likely mechanism for the impaired coupling. PEEPi was equivalent in the two groups before the trial; upon commencing the T-tube trial, weaning failure patients developed higher PEEPi than did the weaning success patients. [We have previously described the phenomenon of hyperinflation during a failed weaning trial (51, 52).]

Neuromuscular coupling during the T-tube trial was constant in the success group and, surprisingly, it increased in the failure group (Fig. 1). This increase in coupling was caused entirely by the increase in ΔP_di_ (∆EA_di_ remained constant). Factors that can affect the accuracy of ∆EA_di_ in reflecting central neural output to the diaphragm include changes in muscle fiber action-potential velocity (98) and nonlinearity of the ΔP_di_ to ∆EA_di_ relationship (11). It is unlikely, however, that either factor was operational in our patients. First, changes in the velocity of muscle fiber action potential vary with diaphragmatic fatigue and temperature and alter the amplitude of the EA_di_ relative to that of central neural output to the diaphragm (98). As noted by Liu et al. (63), these changes are not of a magnitude to explain the temporal differences in ΔEA_di_ observed during a failed weaning trial. Second, amplitude of ΔEA_di_ is linearly related to global diaphragmatic activation up to 75% of P_di_max (11). At the conclusion of the failed weaning trial, the mean ΔP_di_ was only 58.8 ± 5.2% of maximum.

The increase of the neuromuscular coupling during the failed T-tube trial was closely related to the increase in mechanical load on the respiratory muscles: the *r*^2^ between coupling and inspiratory resistance during the failed trial was 0.889 (*P* = 0.016). Several mechanisms contributed to this unexpected and novel finding.

During the failed T-tube trial, there was a progressive increase in the ΔP_ga_-to-ΔP_es_ ratio, implying redistribution of neural output to the respiratory muscles (83) with increased contribution of rib cage muscles to inspiration (59). An increase in rib cage contribution to inspiration can reduce diaphragmatic shortening (28), enabling the diaphragm to act as both an agonist and a fixator (70). As an agonist, the diaphragm contributes directly to generation of tidal volume (70). As a fixator, the diaphragm prevents (or diminishes) transmission of pleural pressure to the abdomen (70). The result is an improvement in diaphragmatic coupling (28, 58) (Supplemental Fig. S2, https://doi.org/10.6084/m9.figshare.11903130).

During the failed T-tube trial there was a progressive increase in expiratory muscle recruitment, as indicated by the progressive increase in the expiratory rise in P_ga_ (79) (Fig. 2). Under loaded conditions, substantial expiratory muscle recruitment extends for up to 200 to 290 ms into the ensuing inhalation (postexpiratory expiratory recruitment) (2, 79). During the last 420 to 660 ms of that inhalation, expiratory muscle activity becomes evident again (preexpiratory expiratory recruitment) (2). Postexpiratory and preexpiratory expiratory muscle recruitment (58) improves diaphragmatic neuromuscular coupling by decreasing abdominal wall compliance (34) and, thereby, reduces inspiratory shortening of the diaphragm (34, 37). A decrease in abdominal compliance can also increase the fulcrum effect of the abdominal contents on the diaphragm (28)—an effect that enhances rib cage displacement by diaphragmatic contraction during inhalation (28). Furthermore, even in patients with severe chronic airflow obstruction (24), postexpiratory expiratory muscle recruitment (58) can improve diaphragmatic coupling by increasing the mechanical advantage of the muscle (11, 56).

The increase in ΔP_ga_-to-ΔP_es_ ratio together with postexpiratory expiratory muscle recruitment (58, 66) suggests that loading on the respiratory muscles during the failed T-tube trial triggered a coordinated action of rib cage, accessory, and expiratory muscles, which, in turn, improved the mechanical advantage of the diaphragm. In addition, coactivation of (inspiratory) rib cage muscles facilitated the action of the diaphragm by reducing the muscle’s velocity of shortening during contraction—a form of functional synergism (21). This irradiation of effort to other muscles groups—or synkinesis—likely resulted from lateral spread of excitation among the upper motor neurons (39). Synkinesis is known to involve recruitment of muscles, even muscles with no biomechanical utility for the task, such as facial muscles (39); indeed, patients performing substantial respiratory efforts during a failed weaning trial commonly exhibit facial signs of distress.

ΔPdi increased progressively during the failed T-tube trial (Fig. 1). When subjected to inspiratory loading, laboratory animals generate an increase in ΔP_di_ achieved by progressively recruiting motor units in a rank-ordered fashion (Henneman size principle) (71, 93, 95). Motor units with slower contraction times (slow-twitch) and greater resistance to fatigue are first recruited. This is followed by recruitment of motor units that have faster contraction times, greater vulnerability to fatigue, and greater capacity to generate tension (71). It is not likely that motor units with greater capacity for generating tension and greater susceptibility to fatigue contributed substantially to improved neuromuscular coupling in patients. In the human diaphragm, muscle fibers linked with motor units possessing the greatest capacity to generate tension and greatest vulnerability of fatigue (namely, Type IIb fibers) constitute only one-tenth of muscle mass (59).

Patients who failed the T-tube trial experienced severe respiratory distress, which is known to cause a release of adrenaline and noradrenaline (76). A positive inotropic effect consequent to catecholamine release, leading to improved coupling, is unlikely. The highest catecholamine levels achieved during a failed weaning trial (1.65 ng/ml) (76) are unlikely to potentiate calcium transients and evoke positive inotropy (17). Moreover, catecholamines can decrease peak force in slow‐twitch fibers by shortening twitch-force duration (positive lusitropic effect) (17). This results in an increased relaxation rate of muscle fibers and may serve to increase the speed of rapidly alternating movements (88) in a tachypneic patient.

### What Was the Ultimate Cause of Weaning Failure?

All of the weaning failure patients experienced severe respiratory distress. We reason that respiratory distress resulted from activation of at least two pathways. The first consists of the bronchopulmonary and respiratory muscle C-fibers that project to the cingulate gyrus (74), an area of the brain involved in respiratory sensation (74). These fibers are activated by intense tidal swings in intrathoracic pressures and by increases in muscle tension (74)—both of which occurred during the failed weaning trial (Fig. 2). The second consists in the activation of the premotor cortex by the increased mechanical load imposed on the respiratory muscles (40, 75).

Despite a progressive increase in respiratory motor output—as manifested by an increase in tidal swings of intrathoracic pressure, rate of phrenic motor neuron activation (increase in ΔEA_di_/T_I_), rate of transdiaphragmatic pressure development (increase in ΔP_di_/T_I_), and increase in the total number of motor neurons being recruited (increases in both ΔP_ga_-to-ΔP_es_ ratio and expiratory rise in P_ga_) – tidal volume remained constant during failed T-tube trials. The resultant imbalance between the increase in respiratory motor output, sensed as corollary discharge (112), and afferent feedback from mechanoreceptors of the respiratory system contributed to respiratory distress (77) through the activation of the premotor cortex (40, 75) and of the cortico-limbic structures (5, 36, 80, 112). The latter structures enhance awareness of homeostatic threats arising within the body and especially the viscera, such as threats that cause pain (5, 77).

Notwithstanding the increase in respiratory motor output, ∆EA_di_ at the end of a failed trial was half of maximum, signifying that reflex inhibition of central neural output to the diaphragm (64) contributed to weaning failure. Several spinal and supraspinal mechanisms have been implicated in the development of central inhibition under loaded conditions (104). We reason that the cortex participates in the recruitment of the phrenic motor neurons during the increased inspiratory loading of a T-tube trial, particularly during respiratory distress when patients are failing the trial; this possibility is supported by a strong body of experimental evidence (30, 40, 43, 44, 60, 64, 73, 84–86, 105, 107, 108).

The spinal and supraspinal mechanisms implicated in the development of central inhibition under loaded conditions include the depressant effect of rising endorphin concentrations (92), increased discharge of group III and IV muscle afferents in forcefully contracting inspiratory muscles (39, 104) and nociceptive phrenic afferents (87). A failing cardiovascular response to increased metabolic demand, a common finding in weaning failure (50), can also trigger inhibition of central activation through a rise in pulmonary capillary pressure (23, 39) and decreased perfusion of the central nervous system (111). The latter two mechanisms are unlikely to have been operational in our patients because none of the T-tube trials were stopped for cardiovascular reasons and no patient exhibited clinical evidence of impaired perfusion of the central nervous system.

The likelihood that a reflex mechanism inhibits central-neural output in patients who fail a T-tube trial is supported by the findings of Fuglevand et al. (38) and Petrofsky et al. (81). These investigations instructed subjects to maintain submaximal contractions of the index finger (38) or adductor pollicis (81) until task failure—the moment in which subjects were unable to generate the target force. Despite subjects’ maximal (perceived) effort, electromyogram (EMG) was reduced at task failure compared with the EMG accompanying maximal voluntary contractions recorded before the exercise run (38). Although some of the reduction in EMG may have been secondary to changes in muscle fiber action potential or changes in the summation of motor unit potentials to produce surface EMG, central neural output to the muscle was also reduced as motor nerve stimulation showed poor voluntary activation (67).

### Critique of Methods/Limitations

ΔEA_di_ was submaximal at the point of terminating the failed T-tube trial. Whether ΔEA_di_ would have remained submaximal during the preterminal phases of loading (asphyxia) if the trial had not been terminated cannot be answered (for ethical reasons). Results from animal studies suggest that respiratory motor output during the preterminal phase of loading does not increase, but instead is followed by abrupt apnea (91, 114).

We cannot be certain that maximum ΔEA_di_ in any given patient was the result of maximal diaphragmatic recruitment (55). This consideration raises the possibility that inhibition of central neural output to the diaphragm at the end of a failed trial may have been underestimated in some patients. This possibility strengthens our conclusion that reflex inhibition of central neural output to the diaphragm contributes to weaning failure.

Inspiratory loading causes a number of nonrespiratory responses, including reductions in high-energy electromyogram power of the contracting triceps brachii (110), reductions in voluntary maximal contraction of leg muscles (110), and reductions in the estimate of somatosensory sensation elicited by mechanical stimulation of the digits (3). Conceivably, these responses are mediated by projections of inspiratory afferents to the somatosensory cortex (4). Whether such a “global” rather than “respiration-specific” central inhibition was responsible for the submaximal EA_di_ at the end of a failed weaning trial remains to be determined.

Direct measurement of the central neural output to the phrenic motor neurons in humans is presently not possible. The central neural output, however, triggers depolarization of phrenic motor neurons and subsequent depolarization of the phrenic nerve and motor units. Provided that neuromuscular transmission and muscle fiber membrane excitability are intact, the pattern of the diaphragmatic EMG will be directly linked to both motor unit recruitment and motor unit firing rate (97). This electrical activity is not directly related to the response of any particular set of respiratory neurons from higher centers but rather is the end-result of neural integration of many sources at both supraspinal and spinal levels (35). It follows that the EA_di_ signal can provide a reliable reflection of phrenic nerve activity (1), and, hence, of the central neural output to the diaphragm (46, 68)—changes in strength of the EA_di_ signal are related to changes in global inspiratory muscle activation during breathing (97). As such, EA_di_ has been used as a robust estimate of central neural output to the phrenic motor neurons (10, 18, 29, 33, 41, 46, 65, 68, 69).

Our acquisition system did not allow us to report EA_di_ in absolute units (µV). Instead, we report EA_di_ as a percentage of the maximum EA_di_ recorded during the study (109). It is well recognized that the amplitude of an EA_di_ signal has a large interindividual variability (7, 13, 27, 90, 106). There are several potential sources of this interindividual variability. Anatomical differences in the relationship between the crural diaphragm and the gastroesophageal junction can influence the position or orientation of the electrode relative to the crural diaphragm (13, 63, 102). Anatomical differences in the diameter of muscle fibers can affect the propagation velocity of depolarization and, thus, the shape and size of the muscle fiber action potential (42). Interindividual variability in the number of fibers in a motor unit (62). Differences between subjects in the filtering properties of the electrode or surrounding tissue (13, 63, 102). Expressing EA_di_ as a percentage of the maximum value of EA_di_ recorded during the study permits a more meaningful comparison of diaphragmatic recruitment between patients and between studies than is achieved by reporting EA_di_ in microvolts.

The diaphragm consists of two separate muscles, costal and crural diaphragm (22). Only the crural diaphragm generates electrical activity recorded with esophageal electrodes (EA_di_) (97). Animal experiments reveal differences in responses of crural versus costal diaphragm to hypoxia, hypercapnia, and panting in dogs (31, 32), but not in rabbits (20). Human subjects and patients exhibit equivalent activity of the crural and costal diaphragm in response to inspiratory tasks (94, 97) [see Fig. 2 in Sinderby et al. (97)].

Reliable calculation of TTdi is critically dependent on an accurate measurement of P_di_max during a combined Mueller-expulsive maneuver (59). Critically ill patients have great difficulty in performing this combined maneuver (55) and are unable to activate completely the diaphragm during a “maximum” maneuver [see Fig. 4 in Laghi et al. (55)]. Underestimation of P_di_max will necessarily produce an overestimate of TTdi. To avoid interfering with patient physiological responses during the T-tube trial, we did not control for changes in lung volume, diaphragm length, or chemical drive during data recording (59, 103).

### Implications for Future Research

If, as we propose, a relative reflex inhibition of central neural output to the diaphragm is a determinant of weaning failure, then strategies to enhance recruitment of respiratory motor neurons should be explored.

Nonpharmacological strategies, such as strength training (16, 39) and pharmacological strategies (39), can increase recruitment of motor neurons. Strength training causes an increase in recruitment of motor neurons much more than it causes an increase in muscle cross-sectional area (16, 39). [Training-associated increase in cross-sectional area reflects an increase in the force-generating capacity of the muscle distal to the neuromuscular junction (16, 39).] Booth and Thomason (16) estimated that cross-sectional area increases by only 0.1% per day with strength training, whereas voluntary force typically increases by 1% per day. These observations suggest that the 20% increase in maximal inspiratory pressure reported by Martin et al. (72) in difficult-to-wean patients following 10 sessions of inspiratory muscle strength training was mainly driven by an increase in recruitment of respiratory motor neurons rather than increases in muscle mass.

Pharmacologic strategies that could increase motor neuron recruitment and, thus, limit the reflex inhibition of central neural output to the diaphragm include upregulation of serotonin and dopamine activity in the central nervous system (39). If the purpose of the inhibition of central neural output to the diaphragm (39) is to protect the respiratory muscles against contractile fatigue (55) (and load-induced muscle damage) (39), then strategies to limit central fatigue might damage the respiratory muscles (59). Particularly troublesome is the study by Bissett et al. (15), in which inspiratory muscle training following extubation caused large improvements in inspiratory strength (suggestive of enhanced muscle recruitment) but was accompanied by an increase in hospital mortality: 12% in the training group versus 0% in the control group. While the challenge of designing and undertaking studies to increase motor neuron recruitment of the diaphragm will be considerable, the scientific motivation for such research is stronger than before.

In summary, patients who failed a T-tube trial displayed diaphragmatic strength similar to that of weaning success patients. The mechanical load during the trial, however, was greater in the failure group than in the success group. The increase in load caused redistribution of the neural output to the respiratory muscles. The result was an unexpected improvement in neuromuscular coupling of the diaphragm during the failed trial. Despite an increase in respiratory motor output at the end of a failed trial, diaphragmatic recruitment was submaximal. In conclusion, diaphragmatic recruitment is submaximal at the end of a failed weaning trial despite concurrent respiratory distress. This finding signifies that reflex inhibition of central neural output to the diaphragm contributes to weaning failure.

## GRANTS

Dr. Laghi received research grants from the National Institutes of Health, VA Research Service, Liberate Medical LLC, and the National Science Foundation. Dr. Shaikh received research grants from the National Science Foundation. Dr. Jubran has received research grants from the National Institutes of Health.

## DISCLOSURES

Dr. Tobin receives royalties from McGraw-Hill for two books published on critical care medicine. The remaining authors have no conflicts of interest.

## AUTHOR CONTRIBUTIONS

F.L., A.J., and M.J.T. conceived and designed research; F.L., H.S., S.L., and A.J. performed experiments; F.L., H.S., and D.M. analyzed data; F.L. and M.J.T. interpreted results of experiments; F.L. prepared figures; F.L. and M.J.T. drafted manuscript; F.L. and M.J.T. edited and revised manuscript; F.L., H.S., S.L., D.M., A.J., and M.J.T. approved final version of manuscript.
